# Anchor-Point Modulation for Multiplanar Hip Torque Generation in Cable-Driven Exosuits: A Geometry-Based Empirical Model

**DOI:** 10.3390/biomimetics11080579

**Published:** 2026-08-13

**Authors:** Avinash S Pramod, Sejun Park, Jeongho Choo, Giuk Lee

**Affiliations:** 1 School of Mechanical Engineering, Chung-Ang University, Seoul 06974, Republic of Korea; avinashsp01@cau.ac.kr (A.S.P.); pmjk22673@cau.ac.kr (S.P.); y0yyht@cau.ac.kr (J.C.); 2 HUROTICS Inc., Seoul 04315, Republic of Korea

**Keywords:** multiplanar assistive torque, hip exosuits, cable-driven systems, anchor-point modulation, torque prediction model

## Abstract

Cable-driven hip exosuits are designed to deliver assistive torques during locomotion. However, most studies focus on assistance in a single plane, even though hip mechanics are inherently multiplanar. This study investigates whether modulation of proximal and distal cable anchor-point locations can systematically alter the distribution of hip assistive torque across anatomical planes. To characterize and estimate three-dimensional torque, we propose a geometric model that incorporates empirically identified correction terms. The model was evaluated using a hip–thigh phantom across 12 anchor configurations and 17 joint-angle conditions, resulting in 204 experimental cases. The results show that anchor-point modulation systematically redistributes assistive torque among the sagittal, frontal, and transverse planes, demonstrating that changes in cable routing can generate distinct multiplanar torque profiles. The model captured major trends and directional characteristics of the torque, with strongest agreement observed for the $M_{z}$ component, for which the mean absolute percentage error ranged from 4.7% to 10.1%. Estimation errors increased under conditions of greater cable wrap, reflecting the influence of torsion and compliance effects not explicitly represented. These findings establish anchor-point modulation as a practical method for achieving multiplanar hip assistance and provide a foundational empirical baseline for extending cable-driven assistance beyond conventional sagittal-plane operation.

## 1. Introduction

Wearable cable-driven hip-assist exosuits are designed to provide coordinated support during locomotion while minimizing distal mass by transmitting actuation remotely through cables [1,2,3,4]. Unlike rigid exoskeletons, soft exosuits transmit assistive forces through flexible textile structures, allowing the actuation system to be positioned away from the assisted joint while reducing constraints associated with rigid joint alignment [5,6]. Existing studies on hip exosuits have predominantly focused on sagittal-plane assistance, particularly hip flexion and extension, because these motions contribute substantially to forward progression during walking [7,8,9,10,11]. Initial studies on cable-driven hip assistance demonstrated the feasibility of applying tensile assistance to the hip via a remote actuation system and established the importance of the cable path in generating the desired hip-extension torque [12].

However, hip mechanics are inherently multiplanar. Normal gait involves coordinated flexion/extension, abduction/adduction, and internal/external rotation across the sagittal, frontal, and transverse planes, respectively [13]. Patients who have undergone hip arthroplasty, as well as those with hip osteoarthritis, often exhibit deficits not only in sagittal-plane moments but also in frontal- and transverse-plane torque generation during critical gait phases [14,15,16,17,18]. Multiplanar assistance is also valuable for clinical cohorts requiring secondary-plane stabilization, including individuals with hip abductor weakness or neuromuscular gait impairments, as well as healthy workers performing asymmetric lifting tasks. Supplying adaptable off-axis torque components alongside primary sagittal assistance directly addresses these multi-axis loading demands. In cable-driven systems, generating multi-axis torques relies on transmission geometry. Because a cable generates force along its line of action, the spatial relationship between the cable and the joint center determines the resulting moment magnitude and direction. Thus, extending cable-driven assistance beyond the sagittal plane requires consideration of how cable routing influences the torque vector rather than only the magnitude of a single torque component.

Recent work has demonstrated the feasibility of using cable-driven exosuits to generate assistance outside the sagittal plane. A soft exosuit designed to provide hip-abduction assistance was experimentally evaluated during walking and demonstrated a reduction in the external knee adduction moment, providing evidence that cable-based actuation can influence frontal-plane motion [19]. This work highlights the potential of cable routing to control the direction of the applied assistive moment and motivates further investigation of the geometric factors that determine the resulting multiplanar torque.

The geometry of a cable-driven system is determined in part by the locations of its proximal and distal anchor points. Changing these locations modifies the cable line of action relative to the joint center and, therefore, changes the effective moment arm. Experimental studies of hip exosuits have shown that the anchor-point position affects force-transmission capability and human-suit stiffness [20]. Similarly, experimental force and torque characterization of cable-driven exosuit actuation has demonstrated that the cable configuration and applied force determine the mechanical output transmitted to the user [21]. These findings indicate that anchor-point configuration is a fundamental design variable when controlling the mechanical effect of a cable-driven exosuit.

The effect of anchor-point configuration is also influenced by the interaction between the exosuit and the wearer. Experimental investigation of lower-limb exosuit anchor points has shown that attachment location can affect human-exosuit interaction, energetics, and biomechanics [22]. In addition, the relationship between cable position and the joint center changes with joint posture and human geometry, which can alter the effective moment arm and the resulting assistance [23]. These dependencies have motivated computational approaches for selecting anchor locations. Simulation-based optimization has been used to investigate the influence of anchor-point positions on muscle activation and metabolic power during hip-extension assistance [24], while personalized attachment-point optimization has incorporated biomechanical and neuromuscular characteristics to determine suitable attachment locations [25].

Recent efforts have explored anchor-point modulation and modified cable routing to influence multiplanar motion in exosuits and related cable-driven assistive devices [26,27,28]. However, out-of-plane moments remain insufficiently characterized across different anchor configurations, and experimental validation of the resulting three-dimensional torque distribution remains limited. Moreover, the effectiveness of multiplanar cable routing is sensitive to user-specific geometry, because the same anchor configuration may redistribute torque differently depending on anatomical alignment [22].

Although the existing literature has established the importance of cable routing and anchor-point placement for exosuit force transmission, human-exosuit interaction, and biomechanical assistance [20,21,22,23,24,25,26,27], the relationship between proximal and distal anchor configurations and the resulting three-dimensional hip torque remains insufficiently characterized. Previous studies have generally focused on force capability, stiffness, energetic effects, muscle activation, or assistance in a predefined direction, rather than systematically quantifying how changes in anchor point redistribute torque across the sagittal, frontal, and transverse planes. This characterization is further complicated by cable curvature, friction, torsion, and compliance in the transmission and human-exosuit interface, which can cause the measured torque to deviate from an ideal geometric prediction [20,21].

To address this gap, this paper presents a geometry-based torque characterization framework that maps proximal and distal anchor-point configurations to multiplanar hip assistive torques. The model was experimentally evaluated using a hip–thigh phantom across 12 anchor configurations and 17 joint-angle conditions. The main contributions of this study are threefold. First, it demonstrates that anchor-point modulation systematically redistributes hip assistive torque across anatomical planes. Second, it proposes a geometry-based model with empirically identified correction terms that captures the major trends and directional characteristics of the measured torques within the tested configuration space. Third, it identifies cable wrap, torsion, and structural compliance as the dominant sources of estimation error under highly wrapped configurations. The remainder of this paper describes the anchor-point configuration strategy, the proposed torque model, the experimental setup, and the corresponding validation results.

## 2. Problem Formulation and Anchor-Point Configuration

**Figure 1:**
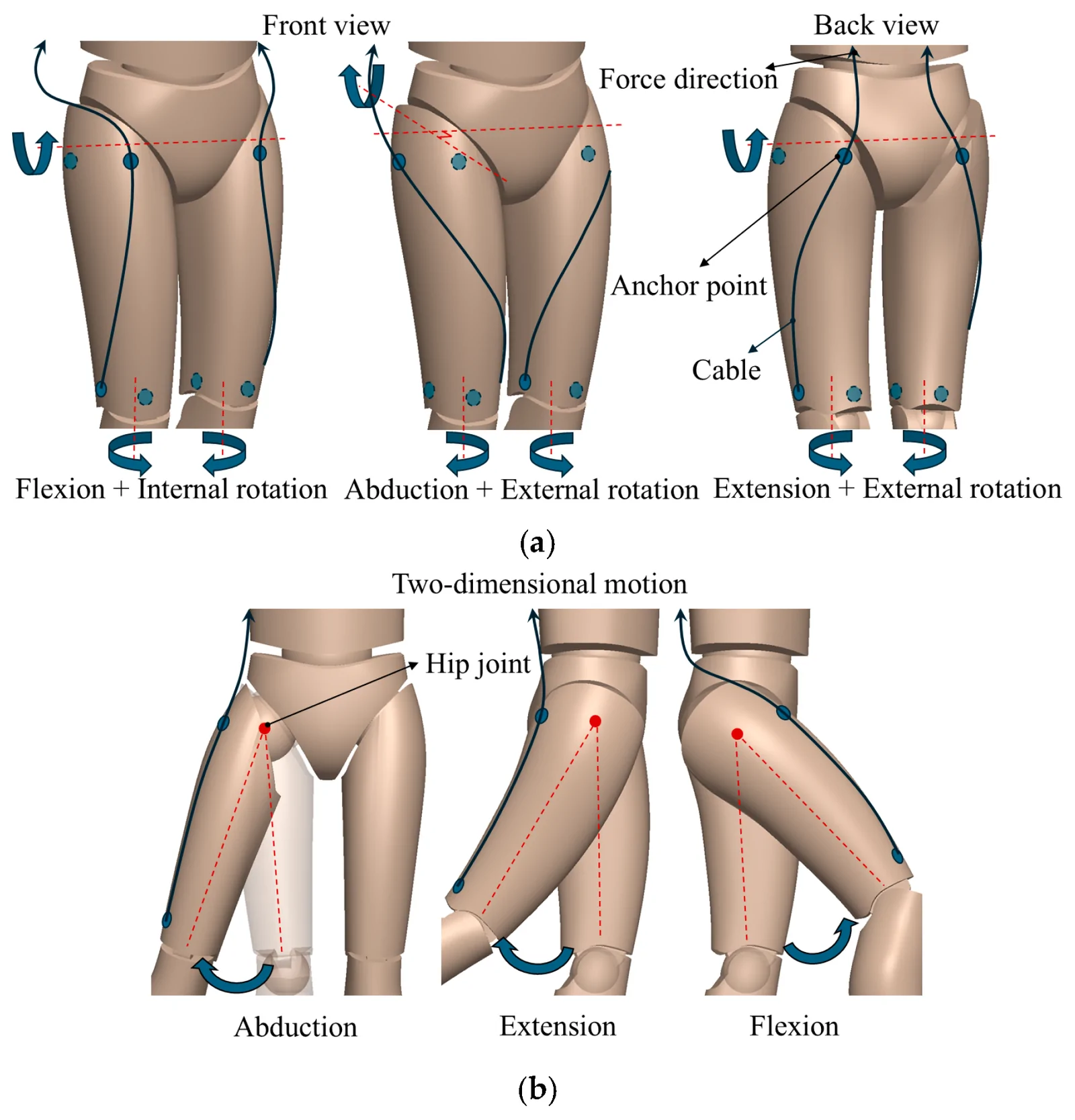
Anchor point configurations used to generate hip elevation under (**a**) multiplanar and (**b**) single-plane conditions.

The objective of this study is to characterize the three-dimensional hip torque generated by different cable anchor configurations. The cable was routed between a proximal attachment on the pelvic side and a distal attachment on the thigh side, as shown in Figure 1. Each configuration was defined by a specific pair of proximal and distal anchor locations. Changing either anchor location, therefore, altered the cable trajectory and the resulting torque generated at the hip. A single cable cannot independently control each torque axis; anchor-point modulation establishes a coupled three-dimensional torque profile that is determined by the cable routing.

Because the continuous space of possible anchor positions around the pelvic and thigh regions contains a large number of possible configurations, the experimental space was discretized into three representative proximal anchor locations and four distal anchor locations. The proximal locations were selected at $0^{\circ }$, $90^{\circ }$, and $270^{\circ }$, while the distal locations were selected at $0^{\circ }$, $90^{\circ }$, $180^{\circ }$, and $270^{\circ }$, resulting in 12 unique proximal-distal configurations. These locations were selected to provide systematic coverage of the circumferential attachment directions around the pelvic and thigh regions and to include both single-plane and multiplanar cable-routing conditions. The resulting configuration set provides a structured basis for evaluating how changes in the spatial arrangement of the proximal and distal anchors influence the measured three-dimensional hip torque.

As shown in Figure 1a, the selected configurations produce multiplanar cable paths, whereas the configurations in Figure 1b represent single-plane conditions. The single-plane configurations provide reference conditions for comparison with the multiplanar cases. These configurations provide a basis for comparing the magnitude and directional distribution of the measured torque under single-plane and multiplanar cable-routing conditions. The anchor configurations were evaluated under different hip postures because the relative position of the cable with respect to the joint changes as the hip rotates. For a fixed pair of anchor locations, the cable trajectory varies with joint posture, thereby changing the corresponding moment arm and torque vector. To capture this effect, each of the 12 configurations was evaluated under 17 joint-angle conditions. The joint angles were varied across the sagittal, frontal, and transverse directions to examine the resulting torque components over the tested configuration space.

The resulting experimental problem can therefore be defined in terms of two variables: the selected proximal-distal anchor configuration and the instantaneous hip posture. For each combination, the generated cable force and the corresponding three-dimensional hip torque were measured. The measured torque was subsequently resolved into sagittal-, frontal-, and transverse-plane components to determine how the anchor configuration and joint posture affect the torque distribution. The following section describes the geometric formulation used to determine the cable path and calculate the corresponding torque.

## 3. Hip Assistive Torque Computation Model

The hip assistive torque generated by each cable-routing configuration is estimated using a geometry-based quasi-static model. The model computes the cable path, moment arms, and tension distribution relative to the hip joint and then combines these quantities to estimate the resulting three-dimensional torque.

### 3.1. Kinematic Modelling of the Cable Path

**Figure 2:**
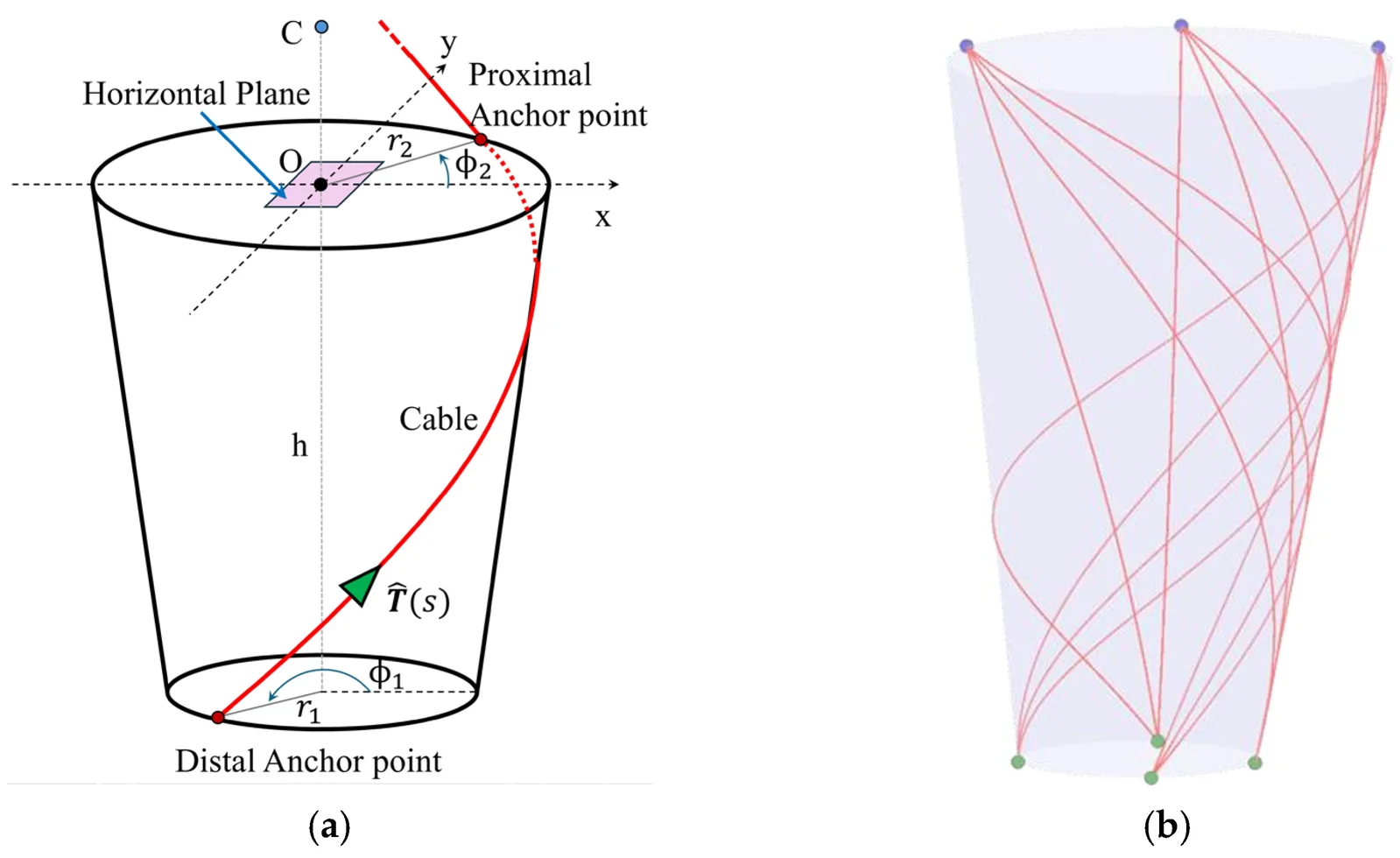
Schematic representation of (**a**) the torque computation model and (**b**) the kinematic cable trajectories between anchor points on the thigh.

To describe the cable path shown in Figure 2a, the model uses the distal and proximal attachment radii ($r_{1}$ and $r_{2}$), the corresponding angular positions ($\phi_{1}$ and $\phi_{2}$), and the vertical separation, h, between the anchors. Here, $r_{1}$ and $\phi_{1}$ denote the radius and angular position of distal anchor, whereas $r_{2}$ and $\phi_{2}$ denote those of the proximal anchor. Hip posture is represented by the joint-angle vector $\Theta ={\left[\theta_{sag},\theta_{fro},\theta_{tra}\right]}^{T}$, corresponding to the sagittal, frontal, and transverse planes. Unless otherwise stated, all trigonometric calculations are performed in radians.

(1) $$\phi_{wrap}=\mathrm{atan}2\left(\sin\left(\phi_{2}-\phi_{1}\right),\cos\left(\phi_{2}-\phi_{1}\right)\right)$$

Based on the cable kinematics, two routing conditions are possible: (i) a straight-line path with zero wrap angle and (ii) a wrapped path with a nonzero wrap angle. Under the zero-wrap-angle condition, the cable path is defined using the position vectors of the distal and proximal anchor points, $R_{A}$ and $R_{B}$, respectively as:

(2) $$R_{A}={\left[\begin{array}{ccc} r_{1}\cos\phi_{1} & r_{1}\sin\phi_{1} & -h \end{array}\right]}^{T}$$

(3) $$R_{B}={\left[\begin{array}{ccc} r_{2}\cos\phi_{2} & r_{2}\sin\phi_{2} & 0 \end{array}\right]}^{T}$$

Under the nonzero-wrap-angle condition, the cable is modeled as a continuous 3D position vector, $R\left(s\right)$, parameterized by the angle s. The radius $r\left(s\right)$ and height $z\left(s\right)$ of the 3D cable path are given by (4) and (5), respectively.

(4) $$r\left(s\right)=\frac{r_{2}-r_{1}}{\left|\phi_{wrap}\right|}\left(s-\phi_{1}\right)+r_{1}$$

(5) $$z\left(s\right)=h\left(\frac{s-\phi_{1}}{\left|\phi_{wrap}\right|}-1\right)$$

Using (4) and (5) the 3D cable path is defined as,

(6) $$R\left(s\right)={\left[\begin{array}{ccc} r\left(s\right)\cos s & r\left(s\right)\sin s & z\left(s\right) \end{array}\right]}^{T}$$

The tangent vector $\hat{T}\left(s\right)$, local curvature $\kappa \left(s\right)$, and principal normal vector $\hat{N}\left(s\right)$ are obtained from the first and second derivatives of $R\left(s\right)$.

### 3.2. Quasi-Static Multiplanar Torque Modelling

Based on the defined cable kinematics, the quasi-static loading is computed by considering the initial cable tension $T_{0}$ and the surface friction coefficient μ. When a tensioned cable comes into contact with the thigh surface, it generates both frictional and normal contact forces.

For the zero-wrap-angle condition, both the normal-force moment, $M_{n}$, and the friction force moment, $M_{f}$, are zero, and the structural torque is determined entirely by the attachment-point moment, $M_{a}$. Accordingly, the baseline attachment moment, $M_{a,base}$, is given as,

(7) $$M_{a,base}=\left(R_{A}^{\left(0\right)}-C\right)\times F_{A}$$

where C is the center of rotation and $F_{A}$ is the input force. Equation (7) therefore defines the baseline moment for zero-wrap condition.

In the nonzero-wrap-angle condition, the effects of surface friction and normal contact force produce a nonuniform cable tension along the cable path. The cable tension, $T\left(s\right)$, is calculated using the Euler–Eytelwein formulation [29], The absolute value in the exponential term is utilized as a simplification for this quasi-static analysis, reflecting the unidirectional nature of the applied cable tension during the steady-state hold phase.

(8) $$T\left(s\right)=T_{0}exp\left(\left|\int_{\phi_{1}}^{s}\mu \frac{R^{\prime }\left(\sigma \right)\times R^{\prime \prime }\left(\sigma \right)}{R^{\prime }{\left(\sigma \right)}^{2}}d\sigma \right|\right)$$

where σ is the dummy variable of integration representing the intermediate angular position along the cable path. The infinitesimal moment produced by the normal contact force along the cable path is given by

(9) $$dM_{n}\left(s\right)=\left(R\left(s\right)-C\right)\times \left(-T\left(s\right)\kappa \left(s\right)R^{\prime }\left(s\right)\hat{N}\left(s\right)\right)ds$$

Similarly, the infinitesimal moment due to the frictional contact force $dM_{f}$, which acts along the local tangent vector and is proportional to surface friction, is given by

(10) $$dM_{f}\left(s\right)=\left(R\left(s\right)-C\right)\times \left(-\mu T\left(s\right)\kappa \left(s\right)R^{\prime }\left(s\right)\hat{T}\left(s\right)\right)ds$$

For the cable-wrap condition, the tangential cable pulling force along the surface is also considered, and the baseline attachment moment is given by

(11) $$M_{a,base}=\left(R\left(\phi_{1}\right)-C\right)\times \left(T_{0}\hat{T}\left(\phi_{1}\right)\right)$$

In addition to the moments generated by cable tension at the hip joint, the hip angles in the sagittal, frontal, and transverse planes alter the relative position of the moment arm with respect to the anchor points. This effect is incorporated into the model by introducing the postural moment term through the hip-angle vector $\Theta^{\left(i\right)}={\left[{\theta_{sag}}^{\left(i\right)},{\theta_{fro}}^{\left(i\right)},{\theta_{tra}}^{\left(i\right)}\right]}^{T}$, and the sensitivity matrix, $A^{*}=\left[A_{sag},A_{fro},A_{tra}\right]$, which characterizes how the baseline torque varies with hip posture.

Because changes in the cable path at different hip angles are not explicitly modeled, the sensitivity matrix compensates for the resulting variations in the computed torque and scales the baseline torque according to hip posture. The entries of the sensitivity matrix and the adjustment matrix are identified empirically. Specifically, the residual torque vector, $M_{residual}$, is obtained by subtracting the theoretically estimated torque from the experimentally measured torque at different hip angles. A linear least-squares regression is then performed in MATLAB R2024a to determine the sensitivity matrix that minimizes the error between the theoretical model and the experimentally measured baseline torque:

(12) $$A^{*}=\arg\underset{A}{\min}\overset{N}{\underset{i=1}{\sum }}M_{residual}^{\left(i\right)}-A{\Theta^{\left(i\right)}}^{2}$$

These diagonal matrices are then multiplied by the calculated baseline moments.

Accordingly, after accounting for nonlinear cable wrap, posture-dependent variations, and empirical corrections, the total 3D assistive torque is expressed as the vector sum of all components:

(13) $$M_{total}=K\left[M_{a,base}+\int_{\phi_{1}}^{\phi_{2}}\left(\frac{dM_{n}}{ds}+\frac{dM_{f}}{ds}\right)ds\right]+A^{*}\Theta$$

Explicit derivation of the nonlinear deformation of the skin under cable tension is beyond the scope of the present geometric formulation. Instead, this effect is incorporated through the wrap-angle-dependent adjustment matrix $K=k_{j}^{\left(comp\right)}\left(\left|\phi_{wrap}\right|\right)$. The posture-sensitivity matrix $A^{*}$ is parameterized as a 3 × 3 matrix composed of three column vectors $\left(A_{sag},A_{fro},A_{tra}\right)$. Each vector contains the specific x, y, and z scaling coefficients that dictate how the sagittal, frontal, and transverse postural angles contribute to the base spatial moment. Furthermore, the wrap-angle-dependent adjustment matrix K is formulated as a 3 × 3 diagonal matrix, $K=diag\left(k_{x}k_{y}k_{z}\right)$. These diagonal adjustment coefficients are empirically identified based on the specific moment component and the discrete wrap angle condition, allowing the model to systematically correct for wrap-dependent localized compliance and frictional dynamics. Through this empirical parameterization, the framework provides a lumped approximation of soft-interface non-linearities and material compliance, avoiding computationally intensive viscoelastic derivations while capturing primary interface behavior under load.

## 4. Experimental Validation

**Figure 3:**
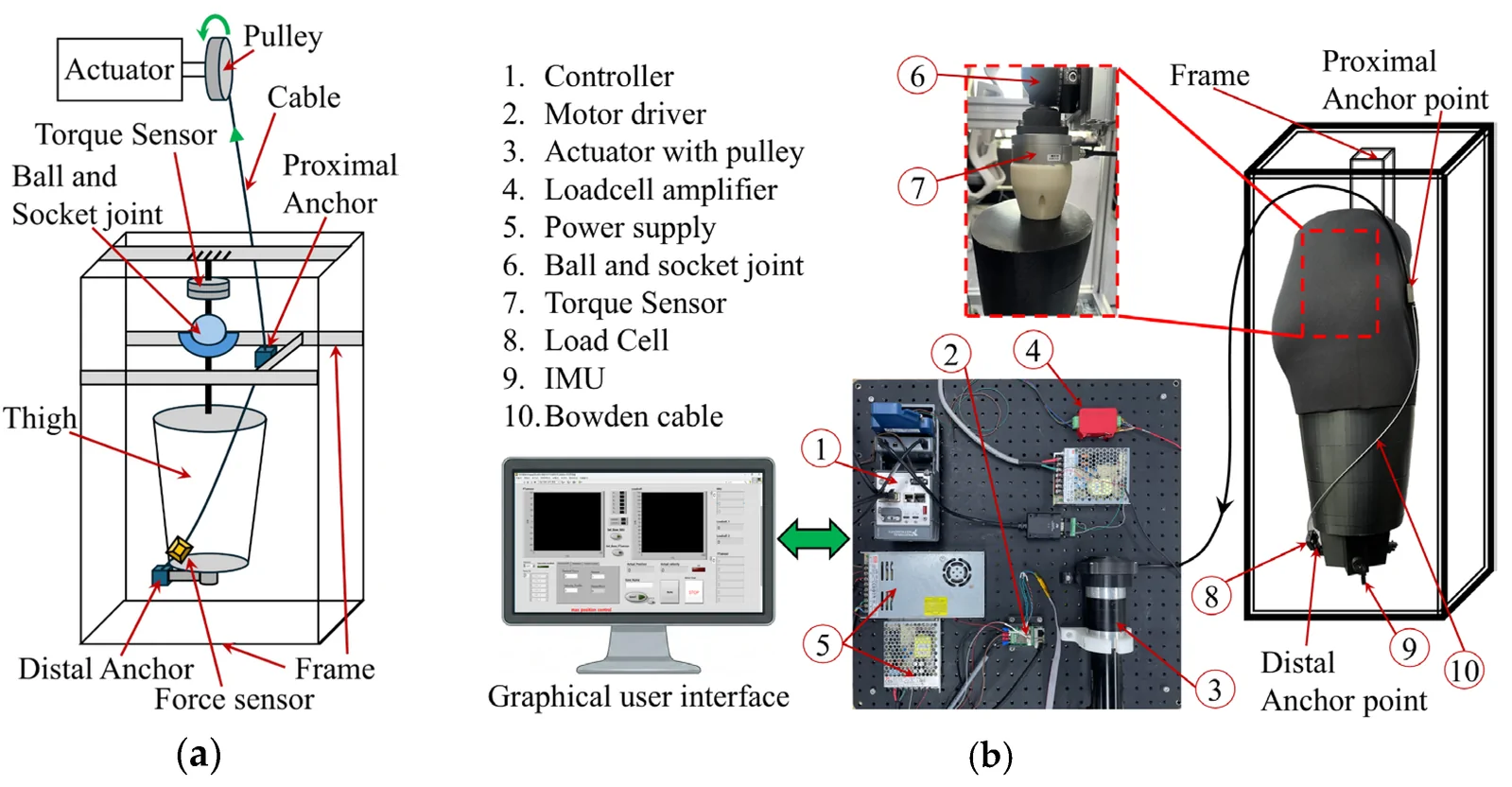
(**a**) Schematic diagram of the test setup; (**b**) experimental testbed used for torque validation, with data acquisition performed using a custom graphical user interface (GUI).

A custom experimental platform was developed to evaluate the proposed torque characterization framework, as shown in Figure 3. The platform integrates a physiologically shaped hip–thigh phantom with a custom cable-driven exosuit test rig.

### 4.1. Hip-Thigh Phantom Hardware Setup

The phantom incorporates a ball-and-socket joint (Thorlabs SL20M, Thorlabs, Newton, NJ, USA) to represent the hip and provide three rotational degrees of freedom. This joint enables controlled positioning and locking of the phantom leg in the sagittal, frontal, and transverse planes. As shown in Figure 3a, the proximal anchor point is rigidly fixed to a dedicated horizontal crossmember on the aluminum structural frame at the pelvic level and acts as a transmission cable guide to hold the cable sheath in place. A 3D-printed thigh segment, based on anthropometric data from an average adult male Korean subject [30], is attached to the joint. A three-axis force/torque sensor (Robotus RFT80-6A02, ROBOTOUS Co., Ltd., Seongnam-si, Republic of Korea) is mounted between the hip joint and the thigh to measure the resulting hip moments. An Ecoflex 00-31 silicone layer is cast around the thigh to emulate soft-tissue deformation under cable loading. A low-friction fabric sleeve is subsequently wrapped over the silicone surface to reduce uncontrolled surface interactions and better isolate cable-routing mechanics. Four distal anchor mounting locations are provided, each incorporating a rotating joint that allows the cable anchor to align with the direction of applied tension. An IMU is attached to the distal center of the thigh to record spatial orientation. The entire phantom assembly is mounted on a rigid test frame.

### 4.2. Exosuit Hardware Setup

The exosuit cable is routed between a proximal anchor near the hip and a distal anchor on the thigh to generate assistive torque about the phantom hip joint as shown in Figure 3b. Cable actuation is provided by a 1-DOF off-board Bowden cable actuator. The actuator consists of a 200 W motor (RE 50, Maxon Motors, Sachseln, Switzerland) coupled to a 181:1 planetary gearbox (GP 62 A, Maxon Motors) (no-load speed: 4900 rpm; stall torque: 7.37 Nm). The cable is wound on a 40 mm radius pulley and fully enclosed within a Bowden sheath to reduce environmental disturbances and prevent unintended contact during loading. A load cell (LSB205, FUTEK Advanced Sensor Technology, Irvine, CA, USA) is integrated near the distal anchor to measure terminal cable tension in real time. The system is controlled using a real-time controller (cRIO-9040, National Instruments, Austin, TX, USA), which acquires sensor data and sends position commands to the motor driver (Gold Twitter, Elmo Motion Control, Petah Tikva, Israel). In each trial, the actuator retracts the cable until the target tension is reached. The position is then held constant, and data are recorded under steady-state conditions to satisfy the quasi-static assumptions of the model.

### 4.3. Experimental Protocol

An experimental protocol was designed to evaluate assistive torque across different cable-routing paths and hip postures. The independent variables included 17 discrete, static joint-angle test postures (1 neutral $\left[0^{\circ },0^{\circ },0^{\circ }\right]$, 8 single-plane postures, and 8 coupled multiplanar postures) centered around neutral offsets rather than a continuous motion curve, spanning the sagittal, frontal, and transverse planes (e.g., [$0^{{}^{\circ}}$, $0^{{}^{\circ}}$, $0^{{}^{\circ}}$], [$0^{{}^{\circ}}$, $0^{{}^{\circ}}$, $10^{{}^{\circ}}$], and [$-10^{{}^{\circ}}$, $0^{{}^{\circ}}$, $0^{{}^{\circ}}$]) and 12 anchor configurations (three proximal and four distal locations), resulting in 204 unique experimental conditions. For each condition, the actuator applied a constant cable tension of 85 N to maintain a taut cable and ensure consistent quasi-static loading, following [31]. Torque data were recorded after the system reached the target tension and steady-state conditions, rather than during the transient pulling phase.

The resulting three-dimensional hip torque was recorded over a duration of 0.5 s. Each condition was repeated across three trials, yielding a total of 612 trials. The dataset was partitioned into training and testing subsets to support parameter identification and evaluate measurement repeatability.

For each anchor configuration and joint-angle condition, two trials were assigned to the training set, and one trial was reserved for testing. The residual torque vector $M_{\mathrm{residual}}$ calculated from the training subset was used to identify the posture-sensitivity matrices $A^{*}$ and wrap-dependent adjustment matrices K using the least-squares optimization in Equation (12). Once determined, these fixed matrix parameters were applied directly to the held-out third trial to calculate $M_{\mathrm{total}}$ using Equation (13). Evaluating this held-out trial independently verified parameter stability and confirmed that the empirical correction terms consistently reflect system characteristics across repeated trials.

## 5. Results

**Figure 4:**
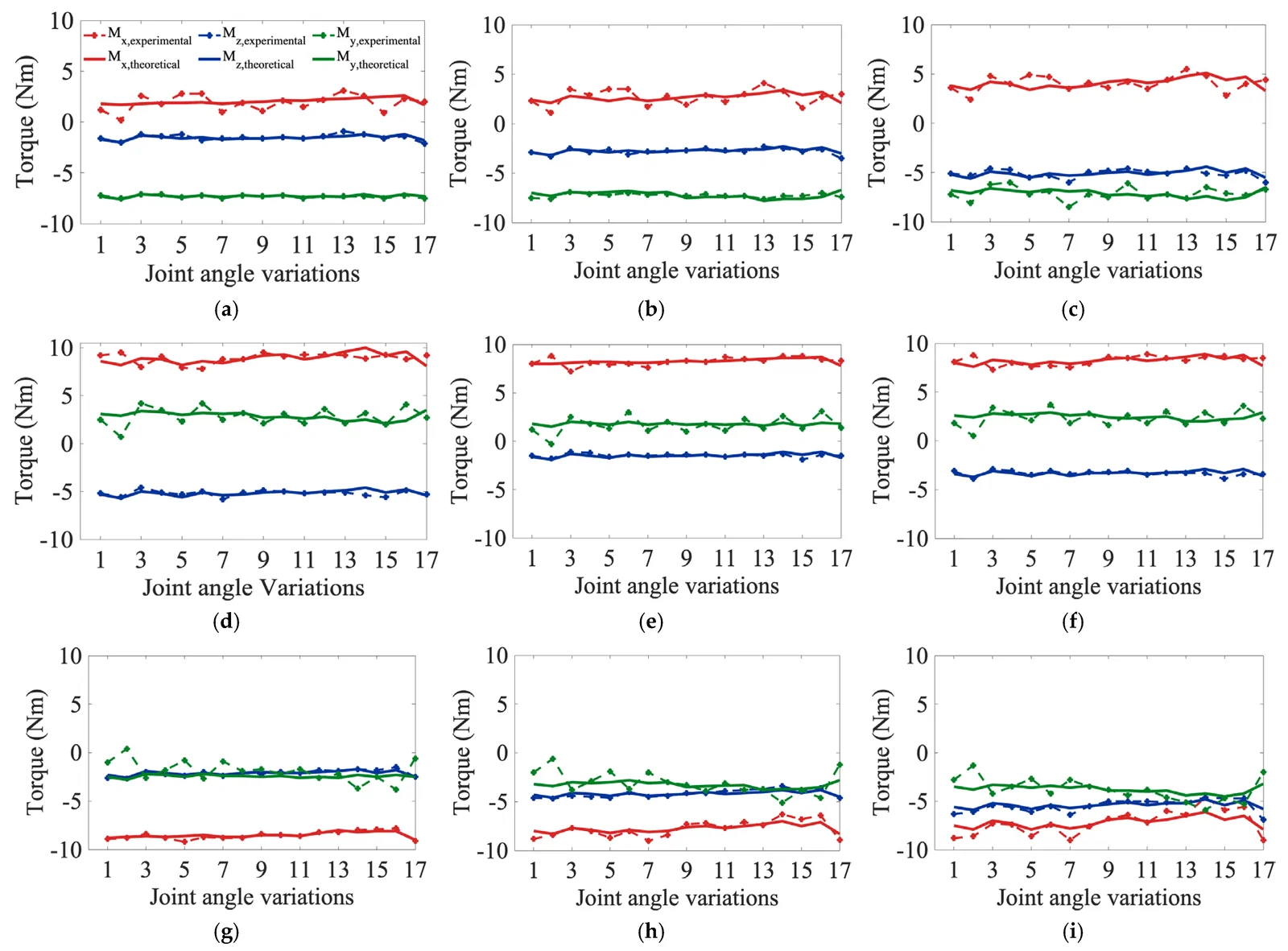
Comparison between theoretical and experimental hip torques across nine anchor combinations: Case 1: (**a**) $\phi_{2}=0^{{}^{\circ}}$ and $\phi_{1}=90^{{}^{\circ}}$; (**b**) $\phi_{2}=0^{{}^{\circ}}$ and $\phi_{1}=180^{{}^{\circ}}$; and (**c**) $\phi_{2}=0^{{}^{\circ}}$, $\phi_{1}=270^{{}^{\circ}}$. Case 2: (**d**) $\phi_{2}=90^{{}^{\circ}}$ and $\phi_{1}=0^{{}^{\circ}}$; (**e**) $\phi_{2}=90^{{}^{\circ}}$ and $\phi_{1}=180^{{}^{\circ}}$; and (**f**) $\phi_{2}=90^{{}^{\circ}}$ and $\phi_{1}=270^{{}^{\circ}}$. Case 3: (**g**) $\phi_{2}=270^{{}^{\circ}}$ and $\phi_{1}=0^{{}^{\circ}}$; (**h**) $\phi_{2}=270^{{}^{\circ}}$ and $\phi_{1}=90^{{}^{\circ}}$; and (**i**) $\phi_{2}=270^{{}^{\circ}}$ and $\phi_{1}=180^{{}^{\circ}}$.

The proposed model was compared with experimental measurements across all 204 test conditions. Figure 4 presents representative comparisons for the nine anchor configurations that produced nonzero cable wrap, allowing an assessment of how effectively the empirical correction terms capture complex interface non-linearities. The remaining three zero-wrap configurations are not shown because their behavior follows the simplified straight-line case, which is captured directly by the baseline model. In each subplot, the x-axis denotes the discrete joint-angle condition, and the y-axis denotes the corresponding torque component.

Across the wrapped configurations, the estimated and measured torques exhibited similar directional changes and overall profile trends. Table 1 summarizes the quantitative error metrics. Among the three torque components, $M_{z}$ showed the most consistent estimation accuracy, with MAPE values ranging from 4.7% to 10.1% across the wrapped cases. Larger errors were concentrated primarily in $M_{x}$ and $M_{y}$, particularly in configurations with larger cable wrap.

The results also show that anchor-point modulation systematically alters the distribution of assistive torque across anatomical planes. In Case 1, shifting the distal anchor within a sagittal-dominant proximal-anchor configuration increased the transverse contribution as cable wrap increased, while preserving the dominant sagittal/frontal behavior.

In Cases 2 and 3, in which the proximal anchor was positioned to favor frontal-plane routing, the same distal-anchor changes redistributed torque between the frontal and transverse directions while leaving the dominant torque direction largely unchanged. These observations demonstrate that anchor placement governs not only the magnitude of the assistive torque, but also its directional composition.

The error patterns were also axis-dependent. In Case 1, the largest deviations occurred primarily in the frontal component, whereas Cases 2 and 3 exhibited larger deviations in the sagittal component. This pattern suggests that estimation sensitivity varies with proximal-anchor orientation. The comparatively lower error in $M_{z}$ is likely attributable to its moment arm being governed primarily by the thigh radius, whereas $M_{x}$ and $M_{y}$ depend more strongly on horizontal offsets, which are more sensitive to cable-induced surface compression and anchor-position compliance.

## 6. Discussion

The experimental results show that the proposed model reasonably captures the dominant trends in the magnitude and directional changes of hip assistive torque across anchor configurations and postural conditions. The combined MAPE, RMSE, and NRMSE values indicate that the framework reproduces the principal behavior of the measured torques while also revealing several physical effects that remain outside the current modeling assumptions.

A key finding is that estimation error is axis-dependent and varies with proximal-anchor orientation. The relatively consistent performance observed for $M_{z}$, compared with $M_{x}$ and $M_{y}$, suggests that torque components governed primarily by the global thigh radius are less sensitive to local deformation and cable-path perturbations. In contrast, the sagittal and frontal components depend more strongly on the horizontal offsets between the hip joint center and the cable path, making them more susceptible to surface compression, anchor compliance, and load-induced geometric shifts. These dependencies confirm that the empirical correction terms are essential for capturing the complex nonlinearities arising from the cable-fabric-silicone interface.

Estimation errors increased under conditions of greater cable wrap, indicating that the proportional redistribution of assistive torque deviates further from the simplified geometric model as wrap severity increases. This trend is consistent with physical effects that are not explicitly represented in the current formulation, including cable torsion between the actuator and the distal anchor, nonlinear contact deformation of the silicone surface, stiction-like friction behavior, and small structural deflections of the phantom under an 85 N load. Because these effects alter the cable path and moment arm in a configuration-dependent manner, they are only partially captured by the empirical correction terms used in this study.

In addition, because several components of the phantom were fabricated by 3D printing and then assembled, structural compliance may accumulate throughout the system and lead to deflection under cable tension. When the actuator applies a cable tension of 85 N, deformation or deflection of the phantom may occur, potentially causing the actual anchor positions to shift relative to those assumed in the model, particularly at the distal attachment points. Because all experiments were conducted at a fixed 85 N load, load-dependent compliance was not evaluated; characterizing the framework across dynamic, multi-load tension profiles is kept as the future scope of this work.

Because the proposed modeling framework is specifically derived for the multi-axis cable routing and soft-interface characteristics of this system, direct comparison against existing models in the literature is not feasible due to fundamental differences in mechanism architecture, anchor geometry, and interface assumptions. Consequently, model assessment in this study focuses on direct validation against physical experimental torque measurements across all test conditions.

Despite the demonstrated efficacy of the proposed characterization framework, this study acknowledges inherent methodological boundaries. The current dataset, while robust for parameter identification and repeatability assessment, does not encompass unseen anchor configurations or postural states. Because the empirical adjustment terms are calibrated within the discrete grid of 12 anchor configurations, the current model functions as a localized characterization tool. Validating the formulation on entirely unseen anchor configurations or independent physical testbeds will be necessary to evaluate broader generalizability, representing an important direction for future research. Additionally, while the stiff phantom provided a controlled setup to isolate cable-routing geometry, living tissue introduces skin movement, active muscle contraction, and localized compression that can cause small mechanical deviations in effective moment arms. Evaluating these tissue interactions through human subject testing represents the immediate next phase of validation. Dynamic human use may introduce additional variations from soft-tissue compliance and relative movement or migration of the skin, straps, and anchor interfaces, which are not represented in the present controlled phantom setup. Furthermore, the present study evaluated discrete static postures within a controlled angular range around neutral to isolate baseline geometric torque shifts. Although larger joint angles ($>30^{{}^{\circ}}$) are physiologically relevant, they induce severe cable wrapping, sliding friction, and complex soft-interface deformations that lie beyond the scope of this quasi-static geometric model. Extending parameter identification to encompass these larger dynamic joint ranges represents a key direction for future work. Finally, this quasi-static, fixed-anchor proof-of-concept serves as a foundational baseline; the transition to dynamic, real-world exosuit deployment necessitates the integration of active mechanisms capable of real-time torque and phase modulation. Such dynamic implementation must concurrently account for the nonlinear interactions and time-varying force profiles characteristic of human soft-tissue deformation during gait.

Despite these limitations, the proposed framework remains useful as an empirical characterization tool. It captures how anchor-point modulation changes the direction and relative distribution of hip assistive torque, which is the central design question addressed in this work. Future refinement of the model should therefore focus on independently measuring and incorporating wrap-dependent compliance, cable torsion, and cable-length variation during multiplanar motion, as well as conducting a formal sensitivity analysis to evaluate torque estimation sensitivity to empirical parameter variations and physical anchor displacement offsets. In practice, since the cable line of action changes gradually with anchor position, minor placement errors due to anatomical differences, body habitus, or suit migration lead to predictable, gradual torque shifts rather than functional failure. This smooth geometric relationship suggests that small anchor-placement deviations may produce gradual rather than abrupt changes in the resulting torque vector; however, the robustness of this behavior under anatomical variability and suit migration remains to be experimentally established.

Beyond model validation, the anchor-point configuration strategy adopted in this study provides insight into the fundamental relationship between cable-routing geometry and multiplanar hip torque generation. The proximal and distal anchor locations were intentionally defined using reproducible circumferential reference positions around the pelvic and thigh regions to systematically evaluate how routing changes influence assistive torque. Consequently, this framework provides a biomechanical mapping between anchor-point location and three-dimensional torque distribution rather than prescribing a single optimal attachment configuration. In future applications, where individual anatomy and specific movement impairments require tailored assistance, this characterization can guide anchor selection to achieve desired multiplanar torques while accounting for coupled off-axis effects. The potential clinical benefits of tailored multiplanar hip assistance, including biomechanical, energetic, symptomatic, and functional outcomes, remain to be evaluated in future human-subject studies.

## 7. Conclusions

This study presented a geometry-based empirical characterization framework for estimating multiplanar hip assistive torque generated through anchor-point modulation in cable-driven exosuits. Experiments conducted using a hip–thigh phantom across 12 anchor configurations and 17 joint-angle conditions demonstrated that variations in proximal and distal anchor locations systematically redistribute assistive torque across the sagittal, frontal, and transverse planes. The proposed model captured the major torque trends and directional characteristics across the tested configuration space, with the largest discrepancies occurring under conditions of greater cable wrap, where torsion, compliance, and contact deformation become more pronounced. These findings identify anchor-point positioning as a key design variable for enabling multiplanar hip assistance and establish an empirical baseline for extending cable-driven exosuit design beyond conventional sagittal-plane assistance. Future work will focus on improving model fidelity by explicitly incorporating cable torsion, wrap-dependent compliance, and cable-length variation during multiplanar motion.

## Figures and Tables

**Table 1:** Comparison of MAPE, RMSE, and NRMSE for Cases 1, 2, and 3.

		$M_{x}$	$M_{y}$	$M_{z}$	$M_{x}$	$M_{y}$	$M_{z}$	$M_{x}$	$M_{y}$	$M_{z}$
Case 1		$\phi_{2}=0^{{}^{\circ}}$ and $\phi_{1}=90^{{}^{\circ}}$			$\phi_{2}=0^{{}^{\circ}}$ and $\phi_{1}=180^{{}^{\circ}}$			$\phi_{2}=0^{{}^{\circ}}$ and $\phi_{1}=270^{{}^{\circ}}$		
	MAPE (%)	24.44	1.31	10.12	27.03	3.57	6.38	15.22	7.18	5.67
	RMSE (Nm)	0.54	0.11	0.21	0.72	0.30	0.22	0.73	0.64	0.32
	NRMSE (%)	25.25	1.55	13.39	26.98	4.15	7.93	18.13	9.15	6.50
Case 2		$\phi_{2}=90^{{}^{\circ}}$ and $\phi_{1}=0^{{}^{\circ}}$			$\phi_{2}=90^{{}^{\circ}}$ and $\phi_{1}=180^{{}^{\circ}}$			$\phi_{2}=90^{{}^{\circ}}$ and $\phi_{1}=270^{{}^{\circ}}$		
	MAPE	5.61	20.25	4.67	3.50	29.54	10.04	4.54	22.40	6.19
	RMSE	0.60	0.70	0.32	0.36	0.57	0.18	0.50	0.63	0.25
	NRMSE	6.69	23.77	6.12	4.38	31.46	12.58	6.06	25.74	7.63
Case 3		$\phi_{2}=270^{{}^{\circ}}$ and $\phi_{1}=0^{{}^{\circ}}$			$\phi_{2}=270^{{}^{\circ}}$ and $\phi_{1}=90^{{}^{\circ}}$			$\phi_{2}=270^{{}^{\circ}}$ and $\phi_{1}=180^{{}^{\circ}}$		
	MAPE	1.50	26.94	6.58	5.50	28.53	4.95	9.03	28.76	5.86
	RMSE	0.17	0.74	0.17	0.50	0.86	0.22	0.77	1.01	0.42
	NRMSE	1.94	30.94	8.02	6.54	26.63	5.38	10.74	26.52	7.55

## Data Availability

The data presented in this study are available on request from the corresponding author.
